# Mini-Factor H Modulates Complement-Dependent IL-6 and IL-10 Release in an Immune Cell Culture (PBMC) Model: Potential Benefits Against Cytokine Storm

**DOI:** 10.3389/fimmu.2021.642860

**Published:** 2021-04-28

**Authors:** Gergely Tibor Kozma, Tamás Mészáros, Tamás Bakos, Mark Hennies, Dániel Bencze, Barbara Uzonyi, Balázs Győrffy, Edward Cedrone, Marina A. Dobrovolskaia, Mihály Józsi, János Szebeni

**Affiliations:** ^1^Nanomedicine Research and Education Center, Institute of Translational Medicine, Semmelweis University, Budapest, Hungary; ^2^SeroScience LCC, Budapest, Hungary; ^3^TECOdevelopment GmbH, Rheinbach, Germany; ^4^MTA-ELTE Complement Research Group, Eötvös Loránd Research Network (ELKH), Department of Immunology, ELTE Eötvös Loránd University, Budapest, Hungary; ^5^Second Department of Bioinformatics and Pediatrics, Semmelweis University, Budapest, Hungary; ^6^Lendület Cancer Biomarker Research Group, Institute of Enzymology, Research Centre for Natural Sciences, Budapest, Hungary; ^7^Nanotechnology Characterization Lab, Cancer Research Technology Program, Frederick National Laboratory for Cancer Research, Frederick, MD, United States; ^8^Department of Immunology, ELTE Eötvös Loránd University, Budapest, Hungary; ^9^Department of Nanobiotechnology and Regenerative Medicine, Faculty of Health, Miskolc University, Miskolc, Hungary

**Keywords:** factor H, complement activation/inhibition, cytokine release syndrome, whole blood assay, COVID-19, immune stimulation, zymosan, anaphylatoxins

## Abstract

Cytokine storm (CS), an excessive release of proinflammatory cytokines upon overactivation of the innate immune system, came recently to the focus of interest because of its role in the life-threatening consequences of certain immune therapies and viral diseases, including CAR-T cell therapy and Covid-19. Because complement activation with subsequent anaphylatoxin release is in the core of innate immune stimulation, studying the relationship between complement activation and cytokine release in an *in vitro* CS model holds promise to better understand CS and identify new therapies against it. We used peripheral blood mononuclear cells (PBMCs) cultured in the presence of autologous serum to test the impact of complement activation and inhibition on cytokine release, testing the effects of liposomal amphotericin B (AmBisome), zymosan and bacterial lipopolysaccharide (LPS) as immune activators and heat inactivation of serum, EDTA and mini-factor H (mfH) as complement inhibitors. These activators induced significant rises of complement activation markers C3a, C4a, C5a, Ba, Bb, and sC5b-9 at 45 min of incubation, with or without ~5- to ~2,000-fold rises of IL-1α, IL-1β, IL-5, IL-6, IL-7, IL-8, IL-10, IL-12, IL-13 and TNFα at 6 and 18 h later. Inhibition of complement activation by the mentioned three methods had differential inhibition, or even stimulation of certain cytokines, among which effects a limited suppressive effect of mfH on IL-6 secretion and significant stimulation of IL-10 implies anti-CS and anti-inflammatory impacts. These findings suggest the utility of the model for *in vitro* studies on CS, and the potential clinical use of mfH against CS.

## Introduction

Cytokine storm (CS), the most intense manifestation of cytokine release syndrome (CRS), is a dysregulated hyperactive immune response characterized by the release of a variety of mediators including but not limited to interleukins, chemokines, interferons, tumor-necrosis factor and other white blood cell (WBC) mediators which, unlike in physiological inflammatory responses, can damage the host. They are also produced as a consequence of severe adverse effect of some monoclonal antibodies and CAR-T-cell therapies (1, 2), and came to the focus of world-wide attention as a contributor to the acute respiratory distress syndrome (ARDS) in Covid-19, as the major mechanism of severe, often fatal outcome of SARS-CoV-2 infection (3–5).

For these reasons modeling CRS/CS *in vitro* is important for better understanding of these adverse conditions and screening of medications against them. It is with this goal that we carried out the studies described here, using a PBMC-culture model of CRS/CS that was found to correlate with *in vivo* features of the disease (6–8). Activation of the first line of immune defense, the complement system, has been known to be a critical contributor to cytokine release by activated immune cells in blood (9, 10). However, the current PBMC-based immunoassays usually utilize culture media supplemented with heat inactivated serum, which excludes getting insights into the role of complement in cytokine release. To fill this gap in *in vivo* relevance, we modified the traditional protocol by supplementing the culture medium with autologous serum. As presented below, this “complement-sensitized” test system enabled the assessment of the role of complement activation in CS/CRS, also highlighting the possible utility of mini-factor H (mfH) against these conditions. In particular, our data suggest that the latter protein, a truncated, recombinant version of the natural complement inhibitor, factor H (fH) (11–15), may have three independent beneficial actions against CS/CRS; suppression of complement activation and complement-dependent IL-6 production, and, stimulation of IL-10 production, a cytokine with anti-inflammatory properties (16–19).

## Materials and Methods

### Materials

For the experiments Dulbecco's phosphate-buffered saline (D-PBS), ethylenediaminetetraacetic acid (EDTA), lipopolysaccharide from Escherichia coli (LPS), Zymosan A from Saccharomyces cerevisiae and the components of complete Growth Medium (cGM, consisting of RMPI-1640 with glutamine, 0.1 mM non-essential amino acids, 50 μM β-mercaptoethanol, 1 mM pyruvate and penicillin/streptomycin) were from Sigma-Aldrich Ltd. (Budapest, Hungary). Ficoll-Paque was obtained from GE Healthcare Bio-Sciences AB (Uppsala, Sweden). AmBisome was purchased from Gilead Sciences Ltd. (Paris, France). The content of the vial, after reconstituting with 12 ml sterile water for injection, contained hydrogenated soy phospholipid (HSPC), 17.75 mg/mL; distearoyl-phosphatidylglycerol (DSPG), 7 mg/ml, amphotericin B, 4.2 mg/ml; cholesterol, 4.3 mg/ml; tocopherol, 0. 05 mg/ml; Sucrose, 75 mg/ml; Sodium succinate, 2.3 mg/ml. The 96-well cell culturing plates (U plate) were obtained from Sarstedt (Nümbrecht, Germany).

### Preparation of Mini-fH

Mini-fH, a polypeptide construct consisting of the 4 N-terminal, ~60 amino acid-containing complement control protein modules (also known as short consensus repeats (SCRs or Suchi repeats) and the two C-terminal SCRs of factor H, was produced in insect cells as described in Refs. (20, 21).

### Mononuclear Cell and Serum Preparation From Blood

Blood was collected from healthy volunteers under ethical protocol TUKEB 15576/2018/EKU and the National Cancer Institute-at-Frederick protocol OH9-C-N046 (in the Nanotechnology Characterization Lab., NCL). Blood anticoagulated with EDTA or Li-heparin (at NCL) was used to purify PBMC using Ficoll Paque gradient density centrifugation according to the procedure described previously (8). Serum was separated by centrifugation of the whole blood at 4°C. Part of the serum was heated at 56°C for 30 min to inactivate complement.

### PBMC Culture

After removing the residual Ficoll and the majority of thrombocytes by washings, PBMCs were washed again with cGM, and 50% autologous serum which was used in the final step for cell suspension. Culturing of PBMCs were done in 250 μl volume in the inner wells of 96-well cell culturing plates (Sarstedt U plate for suspension cells), and each well composed of PBMCs (11-times more concentrated than the original blood, 2.5–5 × 10^6^ cells/well), 50% of normal or heat-inactivated autologous serum and the specified immune activators and complement inhibitors. Plates were incubated in a CO_2_ incubator at 37°C, (except 0-min samples) and samples were obtained in three time points (45 min.: 60 μl, 6 h: 50 μl and 18 h: 140 μl) to prepare supernatants by centrifugation. Aliquots of cell culture supernatants were stored at −80°C until complement or cytokine measurements. For 0-min sampling, cells in cGM and 50% autologous (auto-SE) or heat-inactivated sera (Hi-SE) were immediately processed without any incubation, after diluting them by the solvents of activators (D-PBS) and complement inhibitors (cGM). In another, independent experiment (done at NCL according to the protocol NCL ITA-10 (22) PBMC from 10 healthy donors were incubated for 24 h in cGM supplemented either with 10% heat-inactivated fetal bovine serum (Hi-FBS) or 20% autologous human serum (auto-HS) obtained from the same donor. Cells were stimulated with 20 ng/ml E. coli K12 LPS (PBS served as negative control) and culture supernatants were analyzed by multiplex ELISA for the presence of cytokines (Quansys Biosciences, Logan, UT, USA).

### Complement/Cell Activators and Complement Inhibitors

AmBisome, zymosan and LPS were applied at 2 mg phospholipid/mL, 0.5 mg/ml and 0.5 μg/ml, respectively. To inhibit complement activation EDTA was applied at 20 mM and mfH at 1 *μ*M. Heat inactivation of complement in sera was done by incubation at 56°C for 30 min. In the independent experiment presented in Supplementary Figure 1, in addition to the above stimulants, liposomal doxorubicin (Doxil), phytohemagglutinin and phorbol myristate acetate (PMA)/Ionomycin were applied at 2 mg/ml, 0.1 mg/ml, 5 and 500 ng/ml, respectively.

### Complement and Cytokine Measurements

Complement activation in PBMC supernatant was assessed at 45 min, 6 h and 18 h after starting the incubation by measuring C3a, C4a, C5a, Ba, Bb, and sC5b-9 by a 8-plex chemiluminescence immunoassay (CLIA) (Quansys Biosciences Inc., West Logan, UT, USA), or by individual ELISAs. The levels of IL-1α, IL-1β, IL-2, IL-4, IL-5, IL-6, IL-8, IL-10, IL-12, IL-13, IL-15, IL-17, IL-23, IFNγ, TNFα and TNFβ at 6 and 18 h was measured in the same supernatants by a 16-Plex Human Cytokine kit also from Quansys Biosciences Inc. (West Logan, UT, USA), according to the recommendation of the manufacturer. Data collection was done by “Imager LS” from Quansys, using Q-View Software 3.11 for analysis. The C5a, Bb and sC5b-9 ELISA kits were from TECO*Medical Inc*. (Sissach, Switzerland).

### Data Analysis

The 18-h cytokine values (mean ± SD for *n*=3 different donors) were either given in absolute, or relative terms, by dividing the final concentrations with the respective (0 min) baselines. If values of 0 min measurements were below the quantification limit, the Lower Limit of Quantification (LLOQ) were used for normalization after correction with the dilution. The choice of statistical analyses was based on the fact that the immune activators we used showed substantial differences in activation levels, thus, although the assays were done at the same time, they had to be considered as independent experiments. This ruled out pooling data from the different activator groups for ANOVA. The application of ANOVA was also problematic within the treatment groups because the independent variables were “manipulated within the subjects” inasmuch as cytokine suppression by EDTA could result both from direct cytokine inhibition and indirect complement blockage. Also, we were not “interested” in comparing the complement inhibitors to each other but asked the question of whether the inhibition of cytokine induction was correlating with inhibition of complement, one by one. For these reasons, and because of the low *n*, we used paired *t*-test wherein the dependent variable was compared to baseline for each individual analyte and inhibitor within an activator group. The use of one or two-tailed *t*-tests depended on whether the direction of changes was predictable or not and is specified in the figure legends. The analysis was performed using GraphPad Prism software (San Diego, CA, USA).

## Results

### Complement Activation by AmBisome, Zymosan, and LPS

**Figure 1:**
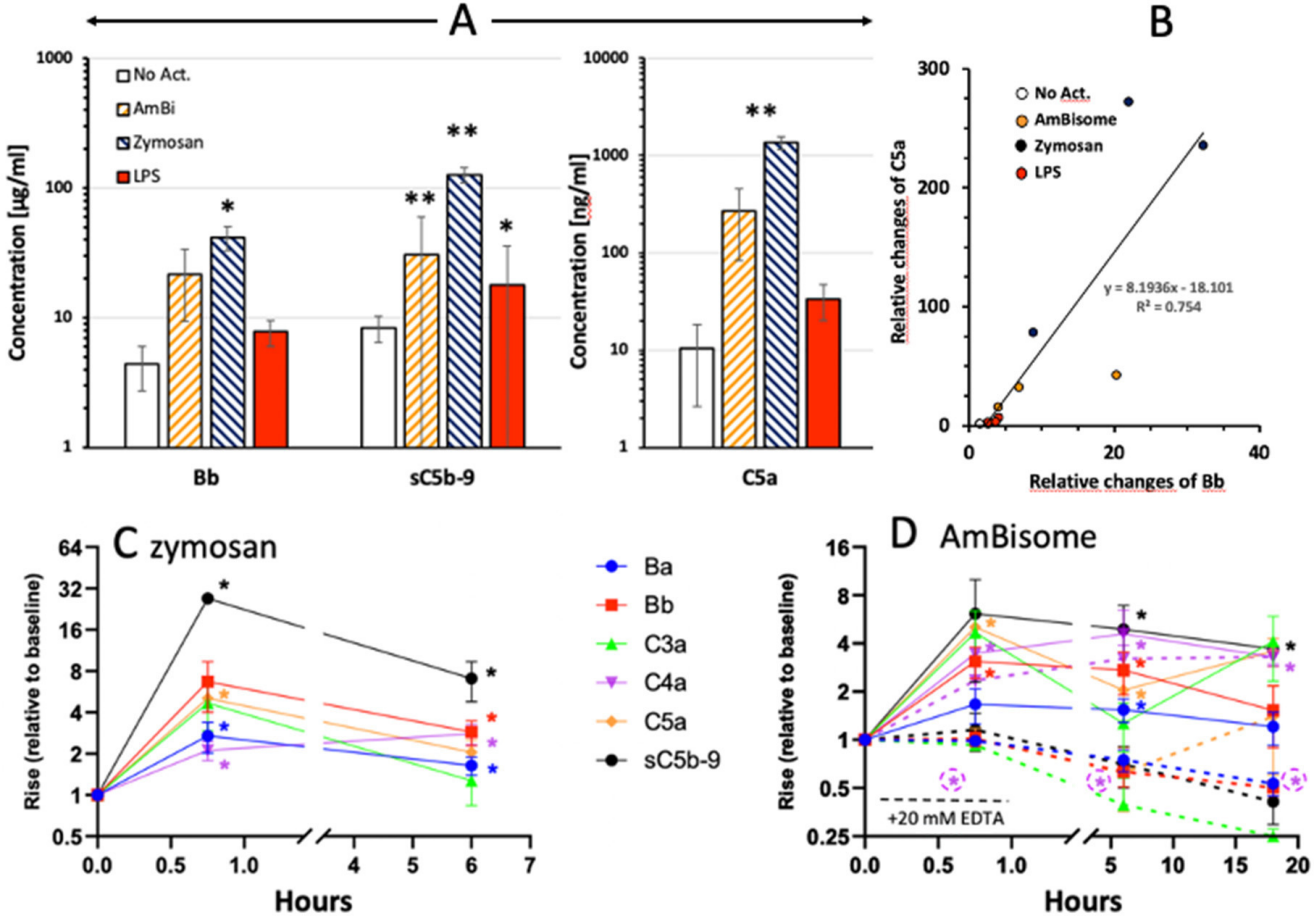
Complement activation by liposomal Amphotericin B (AmBi, 1.98 mg PL/ml), Zymosan (0.5 mg/ml) and LPS (0.5 μg/ml) in PBMC cultures supplemented with autologous serum. **(A)** Columns and error bars represent mean ± SD (*n*=3); ^*^ and ^**^ indicate statistically significant differences comparing to appropriate control (No Act. or baseline) groups, *P*<0.05 or 0.01, respectively. **(B)** Correlation between the individual relative rises (related to 0 min) of C5a and Bb in the samples plotted in **(A)**. Different groups of treatments are represented by different colors (empty: no activation, yellow: AmBisome, blue: Zymosan, red: LPS). Slope shows significant correlation (*P*=0.0002). **(C)** and **(D)**, Similar experiments as in **(A)**, except that the complement activation byproducts were measured by a chemiluminescence immunoassay.

We analyzed complement activation in 2 experimental series, applying individual ELISAs in the first and an 8-plex CLIA in the second. Figure 1A shows the results of the first experiment, indicating significant rises of C5a, Bb and sC5b-9 after 45 min incubation with zymosan, AmBisome and LPS. The simultaneous and correlating rises of C5a and Bb (Figure 1B) indicates that formation of the most effective anaphylatoxin is primarily due to complement activation via the alternative pathway in the case of zymosan and AmBisome. The second series confirmed these changes for zymosan (Figure 1C) and AmBisome (Figure 1D) with the additional information that C3a, C4a and Ba also increased and that the levels of most activation markers decreased after 6 h incubation, except C4a. The effect of 20 mM EDTA is shown for AmBisome (Figure 1D, dashed curves), indicating full suppression of the rise of all activation byproducts, except C4a.

### Inhibition of C Activation in PBMC Cultures

**Figure 2:**
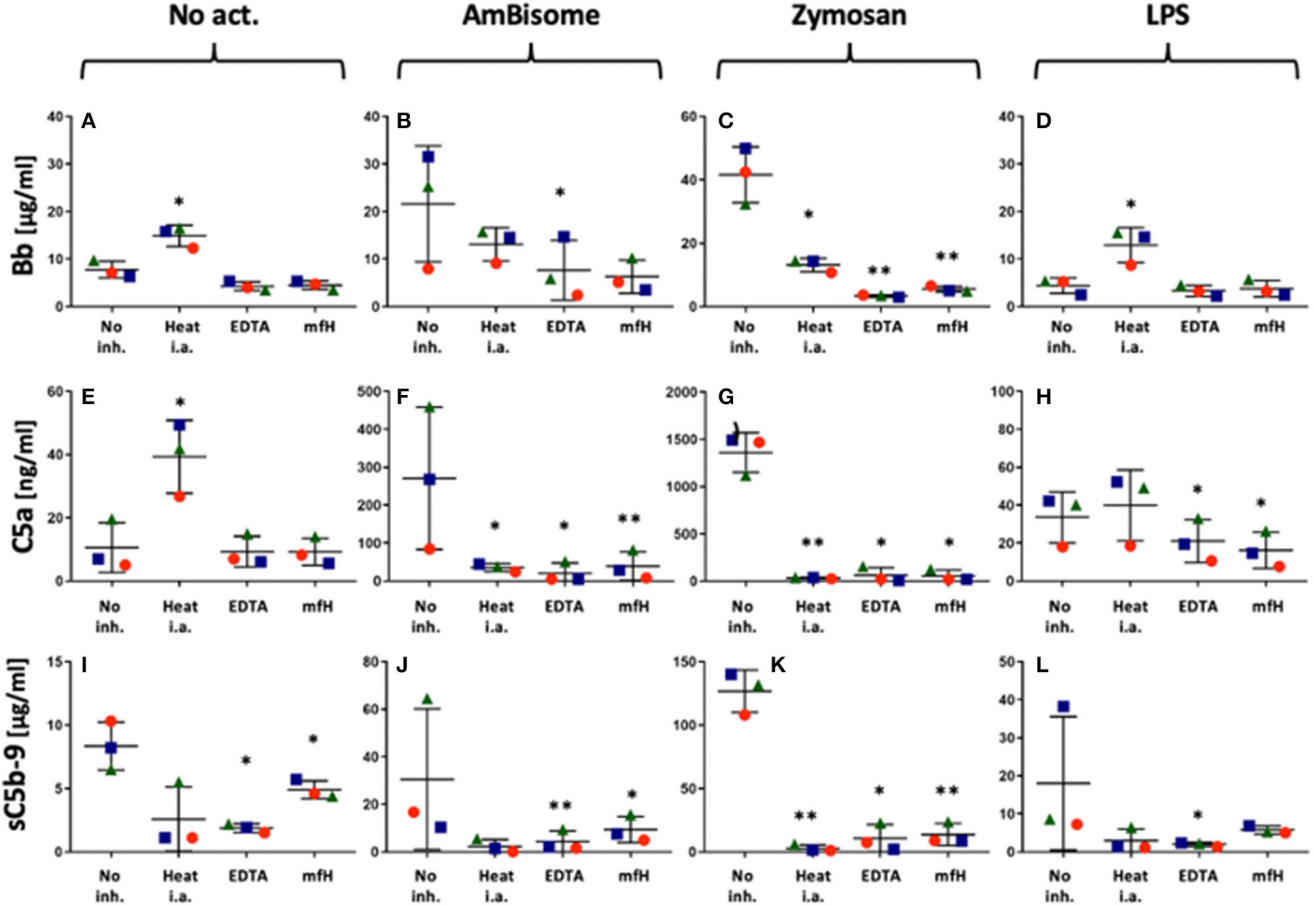
Inhibition of AmBisome-, zymosan- and LPS-induced complement activation (ordered in columns) by different inhibition methods specified on the X axis. No act. and no inh. mean no added activator or complement inhibitor, respectively. On the X axes “Heat i.a.” “EDTA” and “mfH” mean heat-inactivation of autologous serum at 56°C for 30 min, addition of 20 mM EDTA or 1 μM mfH, respectively. Different symbols specify the donors, the bars show the mean ± SD (*n*=3); ^*^(*P*<0.05) and ^**^ (*P*<0.01) indicate statistically significant differences using one-tailed *T*-test comparing the values to the No Act. group.

Figure 2 shows the effects of heat inactivation, EDTA and mini-fH on complement activations by AmBisome, Zymosan and LPS in PBMC cultures, using C5a, Bb and sC5b-9 as endpoints. All these inhibition methods caused major reduction of all activation markers, most efficiently those triggered by zymosan (Figures 2C,G,K). Mini-fH in this case was equally effective as EDTA or heat inactivation (Figures 2C,G,K), exerting > 90% inhibition of complement activation in all three donor PBMC. Interestingly, heat inactivation tended to increase spontaneous C5a and Bb formation in the absence of complement activators (Figures 2A,E).

### Cytokine Release by AmBisome, Zymosan, and LPS in PBMC Cultures: Time Course and Relative Differences

**Figure 3:**
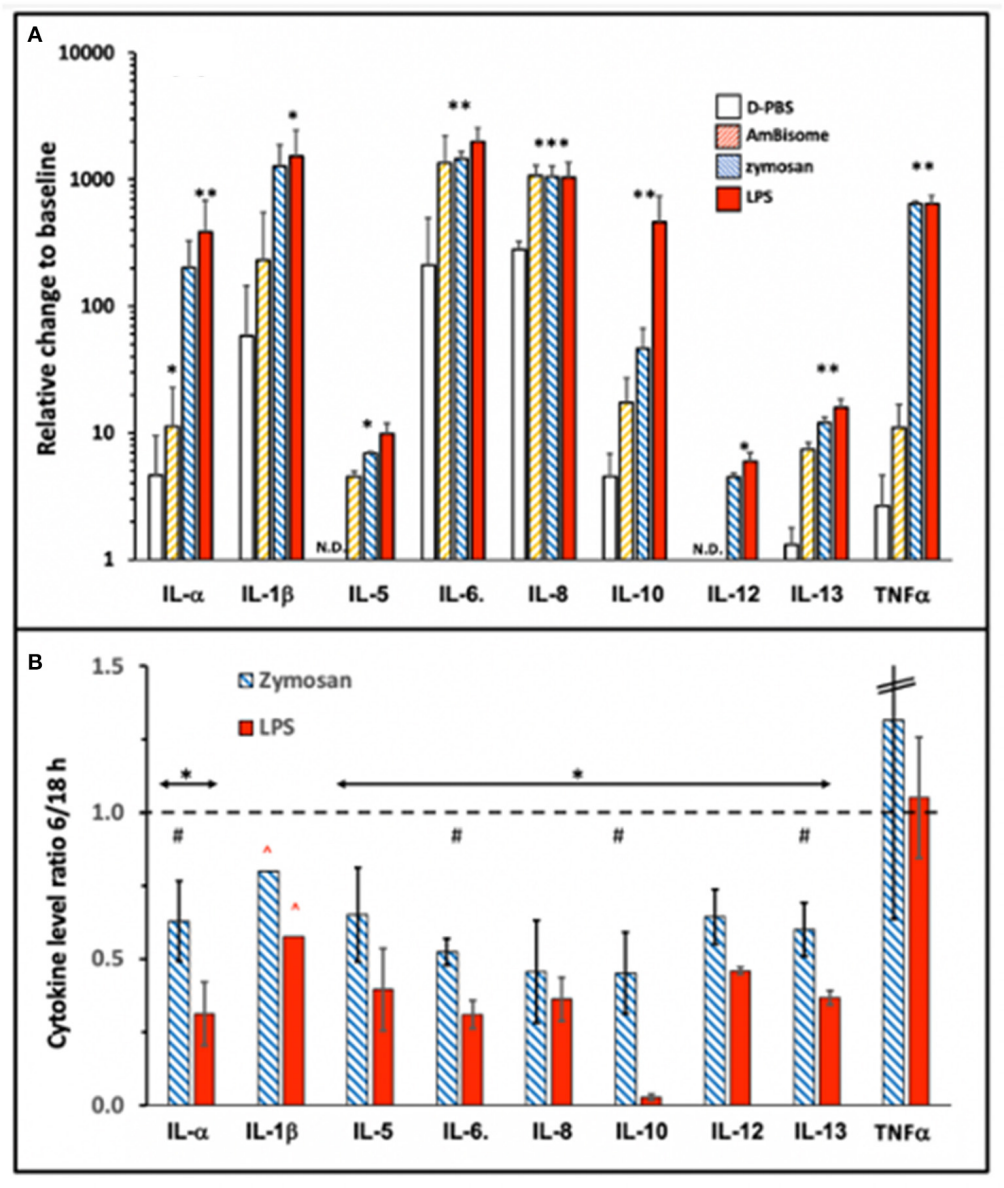
Cytokine induction by immune stimulants that also activate complement. AmBisome, zymosan and LPS were applied at 1.98 mg phospholipid/ml, 0.5 mg/ml and 0.5 μg/ml, respectively, and the PBMC supernatants were analyzed for cytokine levels after 6 and 18 h incubation. **(A)** Shows the cytokine levels expressed as ratios, relative to 0 min baseline at 18 h, while **(B)** shows the ratios of 6 h readings relative to 18 h only for zymosan and LPS. Other details are the same as described for Figures 1 and 2. The bars show the mean ±SD (*n*=3); ^*^ and ^**^ indicate statistically significant increases comparing to D-PBS; *P*<0.05 or 0.01, respectively; #, undetectable rises. **(B)**, ^*^ and # indicate statistically significant decrease using one-tailed *T*-test comparing to 1, or between the two columns, respectively (*P*<0.05); ^∧^ indicates higher real value since data point(s) was/were out of the detection range of the assay.

Among the tested cytokines (see section Methods) IL-2, -4, -15, -17, -23, IFN*γ*, and TNFβ did not show measurable response to the applied immune stimulations (not shown) even after 18h incubation, while 9 cytokines shown in Figure 3 did respond with significant elevations to one or more stimulators. As shown in Figure 3A, the responses relative to 0 min baseline varied between ~5 to ~2,000-fold. Because the 6 h values were generally significantly lower than the 18 h values for all cytokines except TNFα (Figure 3B), 6 h was in the window of dynamic changes for most cytokines, while TNFα could reach plateau already at 6 h. On the other hand, the lack of difference between LPS and zymosan in inducing maximal increase of some cytokines at 18 h (Figure 3A) suggest that the rise of these cytokines reached plateau at this time.

While the cytokine inducing effects of LPS and zymosan were known from previous studies, the effect of AmBisome was surprising since non-PEGylated, highly negative phospholipid vesicles, such as AmBisome, have been known to activate complement but not immune cells for cytokine release. On the other hand, amphotericin B *per se*, can induce cytokines in innate immune cells (23), thus, the membrane-associated antifungal agent might have played a role in the observed cytokine induction by AmBisome, particularly IL-6 and IL-8.

Interestingly, IL-10 was at baseline at 6 h during incubation with LPS (Figure 3B), although it rose to near maximum level at 18 h (Figure 3A). This implies retarded induction of a cytokine that has a negative feedback on the production of inflammatory cytokines (24). As discussed later, this effect may contribute to the strong proinflammatory effect of other stimulants. A further notable observation in Figure 3A is that LPS, whose complement activating effect was the smallest under these conditions, also led to robust cytokine release, just as zymosan, the strongest complement activator. This observation suggests that complement activation was not rate limiting in LPS-induced cytokine release, which is in keeping with differential influence of other controlling factors on the two processes, such as sCD14 and LPS-binding protein (LBP) in serum (25).

To explore the performance of our *in vitro* model at a lower level (10%) of autologous serum, we conducted an additional experiment using PBMCs of 10 healthy donors and tested their cytokine responses to the assay positive control (LPS). As control, we used complete cell culture media supplemented with 10% heat inactivated fetal bovine serum. This study also demonstrated variable, complement-independent induction of most cytokines by LPS except IL-1α and IL-1β, whose production was increased by 10% autologous serum (Supplementary Figure 1).

Taken together, these observations suggest differential regulation of cytokine secretion by complement activation byproducts, which can be studied by adding autologous serum to PBMC cultures. Another important finding in the present study is that PBMC cultures supplemented with autologous serum allow for analysis of cytokines that are known to rise in CRS/CS, including the syndrome observed in severe Covid-19 and immunotherapies such as CAR-T cells (26–28). Moreover, the *in vitro* system affords screening of inhibitory approaches, such as complement inhibition, as shown by the results below. The performance of this model is verified in two laboratories and demonstrates consistent results despite of the use of different percentages of autologous serum. Our study also contributes to the existing knowledgebase emphasizing the predictive capability of PBMC cultures in individualized screening of cytokine responses in human blood donors (29).

### Differential Inhibition of Immune Activator-Induced Release of Cytokines by Different Approaches of Complement Inhibition

**Figure 4:**
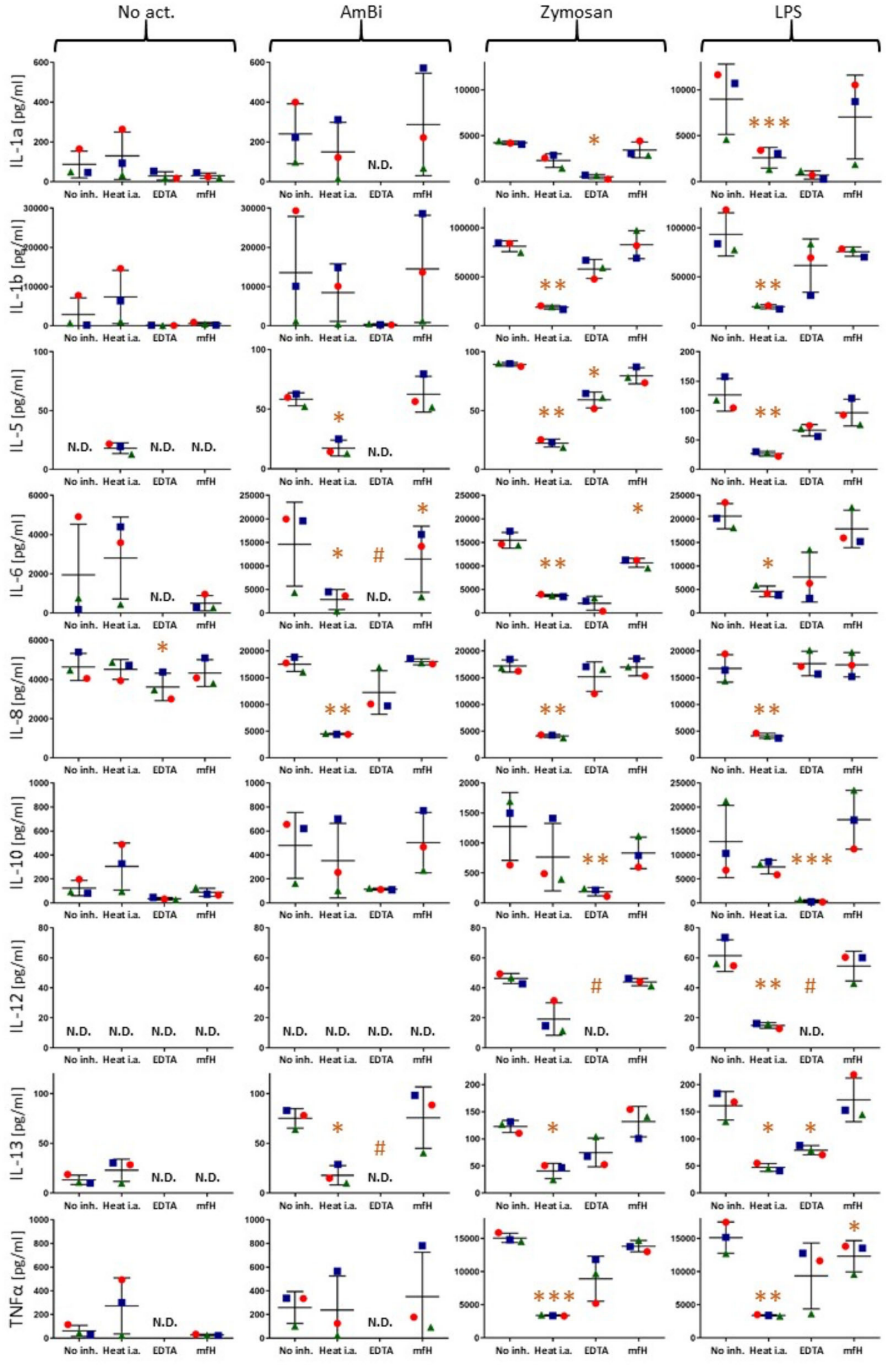
Cytokine levels in PBMC culture supernatants after 18 h activation without any activator (No act.) or with AmBisome, zymosan or LPS, as specified on the top of the figure. No complement inhibition (No inh.), or complement inhibitions of sera by heat inactivation (Heat i.a.), 20 mM EDTA (EDTA), 1 mM mfH are shown on the X axes. Each panel presents data for different cytokines (Y axis labels). The colored spheres, triangles and rectangles specify the three different blood donors. N.D., (non-detectable) means values below the limit of detection (< LLOQ). ^*^(*P*<0.05), ^**^(*P*<0.01), or ^***^(*P*<0.001) imply significant inhibition compared to control (No inh.) by pairwise two-tailed *T* test, # indicates significant inhibition calculated with the LLOQ of the assay.

Figure 4 shows dot plots of individual responses of each responder cytokines following activation with 3 activators (stapled columns) for 18 h at 37°C with or without complement inhibition (inhibitors specified on the bottom axes). In order to show that the individual variation of cytokine responses, when ever seen, is due to differences in individual sensitivity of blood donors rather than measurement (random) error, the three PBMC donors are distinguished by different shapes and colors.

These data provide evidence that inhibition of complement activation can entail inhibition of some cytokines' release. This also means that complement activation contributes to the release of these cytokines, thus, the test system reproduces the clinical observations on the beneficial effects of complement inhibition in CRS/CS, including that observed in Covid-19. A repeat experiment using only AmBisome as stimulant and EDTA, as inhibitor, confirmed the complement-dependent response of IL-1*α*, IL-1*β*, IL-6, IL-10 and TNF*α*, as well as the lack of such response of IL-2 (Supplementary Figure 2).

### Enhancement of Zymosan and LPS-Induced IL-10 Production by Mini-fH at 6 h

**Figure 5:**
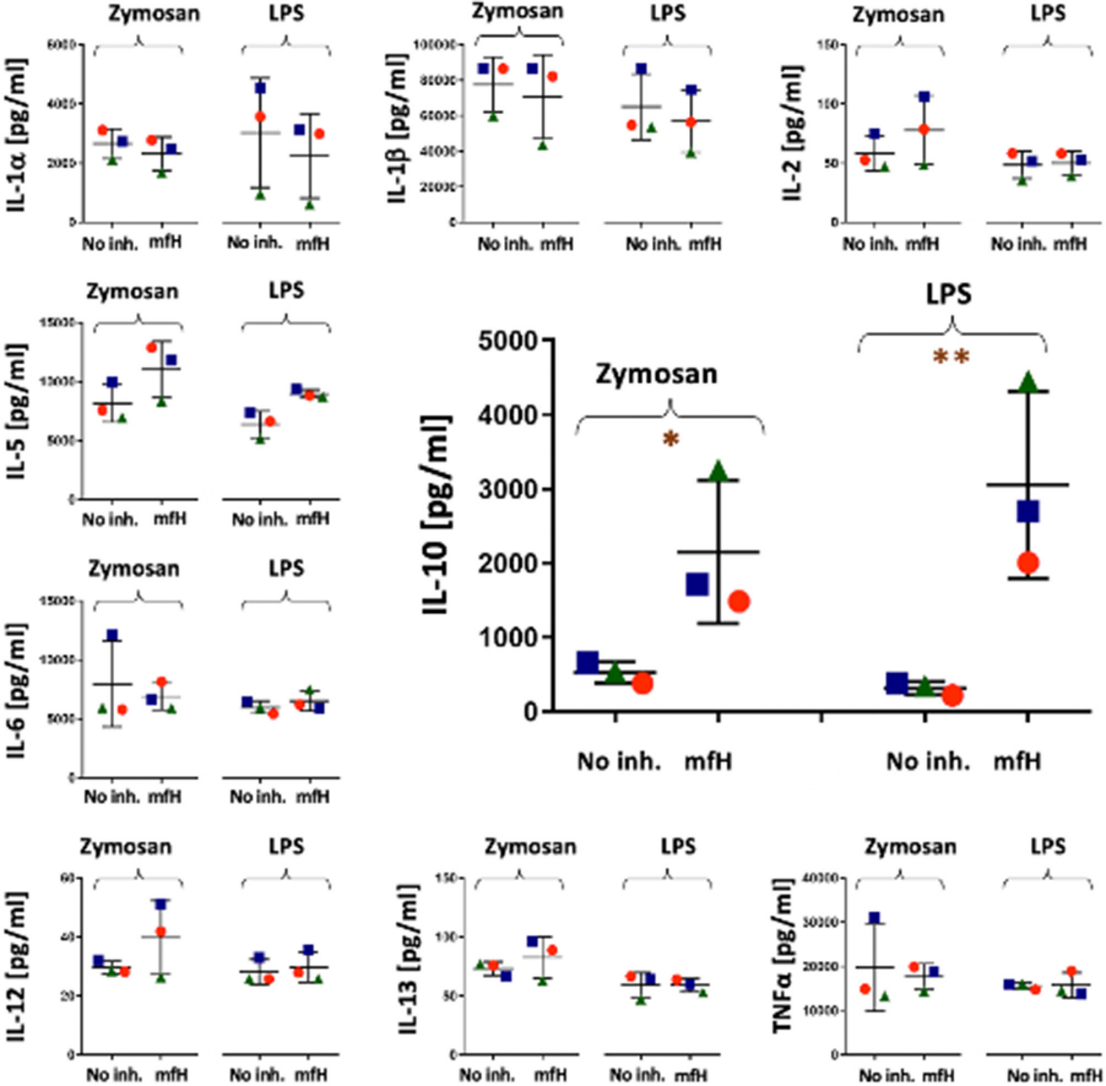
Cytokine levels in PBMC culture supernatants after 6 h activation with zymosan and LPS in the presence and absence of mfH. Each panel presents data for different cytokines (Y axis labels). The colored spheres, triangles and rectangles specify the three different blood donors. The significant stimulatory effect of mfH on IL-10 is enlarged in the middle of the figure. ^*^(*P*<0.05), ^**^(*P*<0.01) imply significant enhancement compared to control (No inh.) by pairwise two-tailed *T* test.

Figure 5, focusing on the inhibition of zymosan and LPS-induced cytokine release by mfH at 6 h, presents an unexpected significant stimulatory effect of this complement inhibitor on IL-10 induction on top of the effects of zymosan and LPS at this time.

## Discussion

### Approaches of Complement Activation and Inhibition

The complement inducers used in this study represent different types of immune stimulants that act both in the humoral and cellular arms of the innate immune response. The liposomal drug AmBisome and the yeast glucan zymosan are potent complement activators whereas LPS is a weak trigger of complement. Both zymosan and LPS are also known for their ability to trigger cell activation via pattern recognition receptors expressed on the surface of immune cells. Specifically, zymosan has been described as a stimulant of TLR 2/6 (30, 31) and another transmembrane signaling receptor, Dectin-1, which collaborates with TLR-2 in NF-*κ*B-mediated cytokine production (32, 33), whereas LPS activates proinflammatory signaling via the TLR4/MD2/CD14 receptor complex (25). It is currently unknown whether AmBisome can trigger the activation of pattern recognition receptors on the surface of the immune cells, although amphotericin B, alone, can do that (23).

Since there was a major difference between the complement activating powers of AmBisome and zymosan, using these two activators enabled us to dissect the significance of complement activating power in inducing cytokines.

Among the tested complement byproducts, C5a, a cleavage product of C5, is a potent proinflammatory anaphylatoxin in the fluid phase; Bb, a cleavage product of factor B whose rise in the fluid phase indicates the involvement of alternative pathway in complement activation; and sC5b-9, also in the fluid phase, provides an indirect measure of membrane attack complex (C5b-9) deposition on cell membranes, entailing cytotoxic pore formation.

The inhibition of complement activation in our study was achieved by EDTA, heat inactivation and mfH, each having different mechanism of action. EDTA prevents the Ca^++^/Mg^++^-dependent buildup of classical and alternative pathway C3 convertases, heat treatment entails the formation of IgG and other protein aggregates and anti-complementary C1 and C1s (34–36) and mFH is a clinically relevant complement inhibitor, a ~373 amino acid-containing, ~42 kDa MW recombinant protein that contains 6 SCRs from fH, the most effective inhibitor of alternative complement activation in plasma (37). The first 4 SCRs on its N-terminal bind to C3b and exert decay accelerating activity on the alternative pathway C3 convertase (C3bBb) and cofactor activity for the C3b cleavage by factor I. The C-terminal 2 SCRs, corresponding to fH 19, 20, bind to C3b fragments (iC3b and C3d) and polyanions (glycosaminoglycans or sialic acid) on host cell membranes. This triple targeting provides a unique, therapeutically valuable defense against complement activation on host cells. Despite a 70% reduction in size relative to fH, mfH extends the functional spectrum of fH outperforming it in a model of paroxysmal nocturnal hemoglobinuria (12). Mini-fH was also shown to protect against experimental glomerulopathy (13, 14) and its phosphatidylinositol-derivative, anchored to endothelial cells, mitigates organ rejection in a porcine xenotransplantation model (11).

### Complement Activation and Inhibition in PBMC Cultures

As expected, we obtained significant rises of all complement activation markers in the supernatant of PBMC cultures incubated with AmBisome, zymosan and LPS, validating the approach of supplementing the tissue culture medium with intact serum. The power of activation decreased in the order zymosan > AmBisome > LPS, although this order does not reflect on biological potency to activate complement since, being a pilot study, the concentrations of activators were chosen on the basis of literature data without attempt to achieve equipotency either in complement activation or cytokine release. Accordingly, the fact that LPS was the least effective complement activator at 0.5 μg/ml is in keeping with earlier data showing major complement activation by LPS (in rat serum) only at 0.5 mg/ml (38).

The effective suppression of all these complement cleavage products by all three approaches of complement inhibition also validates the model inasmuch as it shows that the applied 50% serum provided sufficient dynamic window for the changes to allow statistical analysis of inhibition. The comparison of the effect of 10% autologous serum vs. 50% for the case of LPS-induced IL-1*α* and IL-1*β* (Supplementary Figure 1) also confirmed the essential role of intact serum in cytokine release, and the increase in absolute amounts of these cytokins following LPS stimulation is consistent with the 5-fold greater amount of serum in the case of 50% serum (Supplementary Figure 1 vs. Figure 3A). This proportionality suggests that cytotoxicity by intact (non-heat inactivated) autologous serum does not interfere with quantitative evaluation of cytokine induction, a presumption consistent with that heat inactivation of fetal calf serum is not required for *in vitro* measurement of lymphocyte functions (35).

There were also some unexplainable findings in our complement studies. One was the stimulation by heat-inactivated serum of Bb rise in LPS-treated serum (Figure 2D) and C5a rise in untreated serum (Figure 2E). These observations need confirmation and further studies to understand, just as the massive rise of Ba by zymosan (Figure 1C) and Ca^++^-independent rise of C4a by AmBisome (failure of 20 mM EDTA to block it, Figure 1D). The biological relevance of the latter observations is not clear at this time, but based on available information, some of these changes may be beneficial, since C4a, the third anaphylatoxin (39) was shown to interfere with C5a actions and to have antimicrobial activity (71, 72), and Ba, too, has been shown to have indirect anti-inflammatory properties (40–42).

### Complement-Dependent Cytokine Production in PBMC Cultures

PBMC is known to consist of lymphocytes, monocytes and dendritic cells, all expressing anaphylatoxin receptors (ATRs). Unstimulated T cells express C5a receptor (C5aR) only at a low basal level; the expression of this receptor is strikingly up-regulated upon activation of T-cells (43). It has also been shown that there is strong interaction between TLR and ATR signaling (43), mutually enhancing each other's cytokine inductive effects. In one example of such cooperation, Zhang et al. reported striking rise of plasma IL-6, TNFα and IL-1*β* in decay-accelerating factor (DAF)-deficient mice treated with LPS and zymosan. In this model, the lack of membrane complement inhibitor, DAF, sensitized the animals for anaphylatoxin liberation, and, hence, C3a-C5aR signaling (31). In another example, wild-type mice co-treated with TLR ligands and cobra venom factor, a potent complement activator, significantly increased cytokine production, which was accompanied by increased mitogen-activated protein kinase and nuclear factor-κB (NF-κB) activation in the spleen. These *in vivo* results suggest therefore synergistic ATR and TLR stimulation as an underlying mechanism of cytokine storm.

The efficacy of complement inhibition in attenuating cytokine induction in the present study was shown by near full suppression of IL-1*β*, IL-5, IL-6 and TNF*α* by EDTA and/or heat treatment. In case of IL-6 these results are in keeping with earlier observations on major IL-6 response to the infusion of liposome-encapsulated hemoglobin in rats (45), a treatment that led to massive complement activation under the applied conditions (46). Although Ca^++^ binding by EDTA could inhibit cytokine production independently from complement inhibition, the paralleling, and mostly correlating inhibition of these cytokines' secretion by the two fundamentally different approaches of complement blockade can most easily be rationalized by their common effect, complement inhibition.

**Figure 6:**
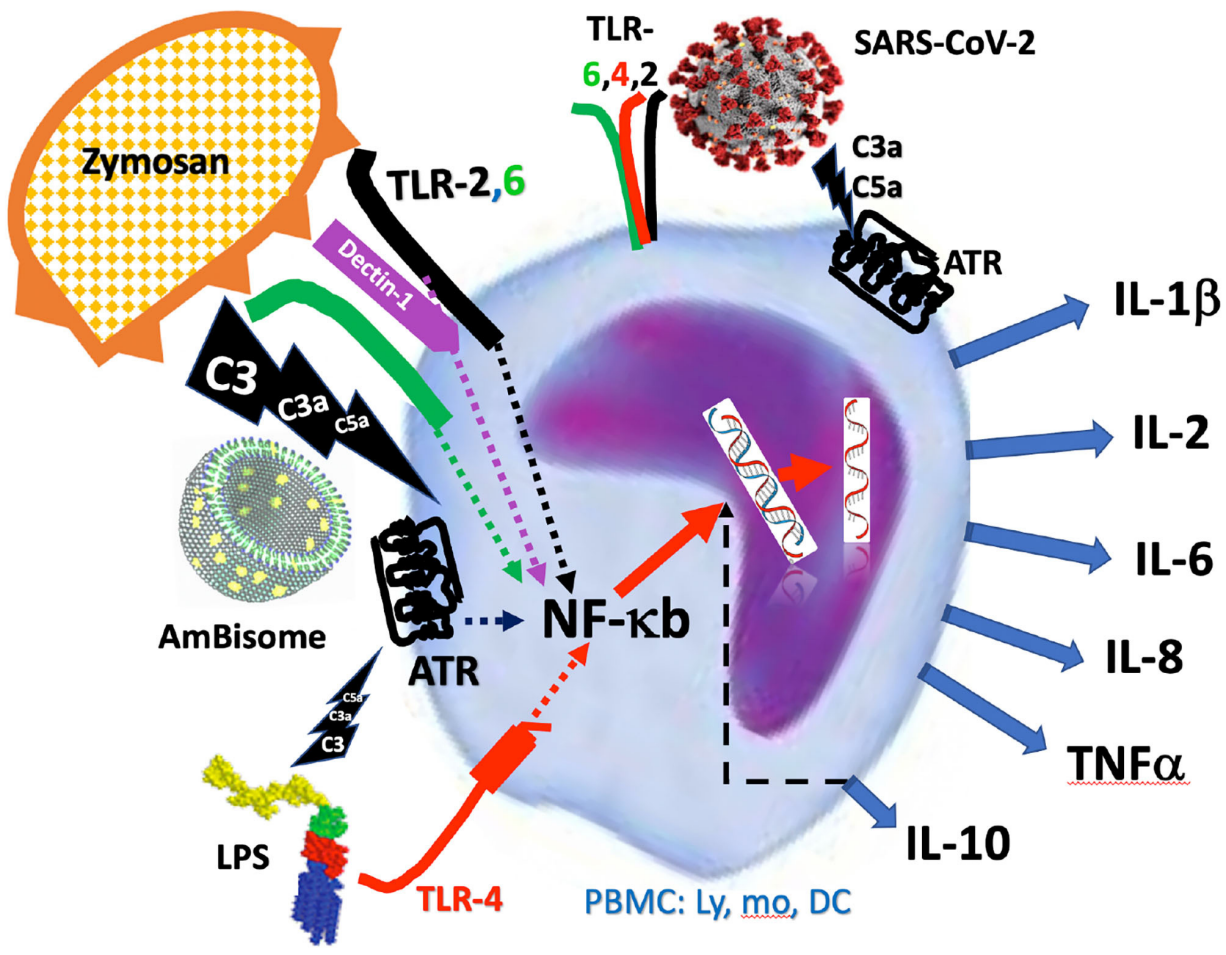
Schematic illustration of activation of PBMC by zymosan, AmBisome and LPS *via* simultaneous engagement of anaphylatoxin and Toll-like receptors (ATR, TLR). The intracellular signaling of activator-receptor binding merges into NF-*κ*b-mediated *de novo* transcription of inflammatory cytokines, explaining the complement inhibition-sensitive production of cytokines. The figure also reminds that the SARS-CoV-2 may also induce cytokine release *via* additive, or synergistic stimulation of both the ATR and Toll-like receptors, making the process complement- dependent.

The scheme in Figure 6 illustrates the above delineated relationships among different activation pathways *via* which zymosan, AmBisome and LPS might have triggered the release of cytokines from responsive immune cells *via* ATRs and TLRs and other pattern recognition or danger signal receptors (47–49). The fact that inhibition of complement also inhibited or reduced the production of some cytokines suggests that the ATR-mediated activation cooperates in these cytokines' release, permitting adding upon or synergizing with cell activation via other channels. However, if a trigger mechanism is overwhelming, there is no need for collaboration with other activation channels. In other words, the efficacy of signal transduction via these channels may represent a spectrum, depending on a variety of factors, and the cells' response may reflect a summation of all concurrent input signals. This “double hit” hypothesis, developed for nanoparticle-induced hypersensitivity reactions (50, 51), is illustrated in Figure 6. It shows that all three immune activators tested in this study trigger at least two activation channels with varying efficacy one being the ATR channel.

### Relevance for COVID-19

Considering the mounting evidence of a critical role of complement activation and anaphylatoxins in the CS in Covid-19 and the efficacy of complement inhibitors in attenuating the disease (52–65), the complement dependence of cytokine release in our PBMC assay highlights the possible clinical relevance of the model for Covid-19 therapy. Infact, the cytokines that were found to be induced by the complement activators, particularly IL-6 and TNFα, are among those typically elevated in Covid-19 (66–69). The inhibitory effect of mfH on IL-6 release (Figure 4) looks promising, as mfH is a druggable protein. The finding is consistent with that mfH is an alternative pathway inhibitor and SARS-CoV-2 activates complement via the alternative pathway (65). The observation that AmBisome was an effective activator of cytokine release is notable because it mimics viruses in terms of bilayer structure and size [80–90 nm], and it too activates complement via the alternative pathway (44). Therefore, it may represent a safe and simple model for studying the innate responses to CS-inducing viruses, such as SARS-CoV-2. Figure 6 highlights the hypothesis that the SARS-CoV-2 may induce cytokine storm via additive, or synergistic induction of both ATR and TLR-mediated intracellular signaling.

## Outlook

Our experiments suggest the utility of non-heat inactivated autologous serum-containing PBMC assay in studying the mechanism and pharmacological sensitivity of CS in general, and, in Covid-19, in particular. Observations in this model point to the possible use of mfH, or similar SCR-based complement inhibitors against pathologies triggered by the excessive cytokine release. Although the inhibition of IL-6 by mfH was relatively small, this study was a pilot exploration of efficacy without attempt to establish dose-effect relationship or pursue other aspects of drug development. It should be noted in this regard that the stimulating effect of mfH on IL-10 at 6 h is another promising observation, since IL-10 is an anti-inflammatory cytokine known to limit tissue damage in chronic severe inflammations (16, 18, 19). Furthermore, the clinical efficacy of convalescent plasma has been suggested not to be due only to neutralizing antibodies, but also to the presence of innate inhibitors of inflammation, including soluble complement inhibitors, such as fH (70).

Being a small recombinant protein with proven efficacy in other diseases (11–15, 20), mfH offers a new strategy against CS in combination with other drugs and treatment modalities, obviously after intense preclinical analysis of efficacy and safety. Its use also draws attention to the potential use of fH and/or other SCR constructs in overcoming the fatality of diseases associated with CS, such as Covid-19.

## Data Availability Statement

The original contributions presented in the study are included in the article/Supplementary Material, further inquiries can be directed to the corresponding author/s.

## Author Contributions

All authors listed have made a substantial, direct and intellectual contribution to the work, and approved it for publication.

## Conflict of Interest

GK, TM, and JS were employed by SeroScience LLC, and MH by TECOdevelopment GmbH. The remaining authors declare that the research was conducted in the absence of any commercial or financial relationships that could be construed as a potential conflict of interest.
