# Supplementary material for: Mini-Factor H Modulates Complement-Dependent IL-6 and IL-10 Release in an Immune Cell Culture (PBMC) Model: Potential Benefits Against Cytokine Storm
Source: Front Immunol. 2021 Apr 28;12:642860. doi: 10.3389/fimmu.2021.642860 (PMC8113956; doi:10.3389/fimmu.2021.642860)
Supplement: Supplementary file 1 [file Table_1.docx]

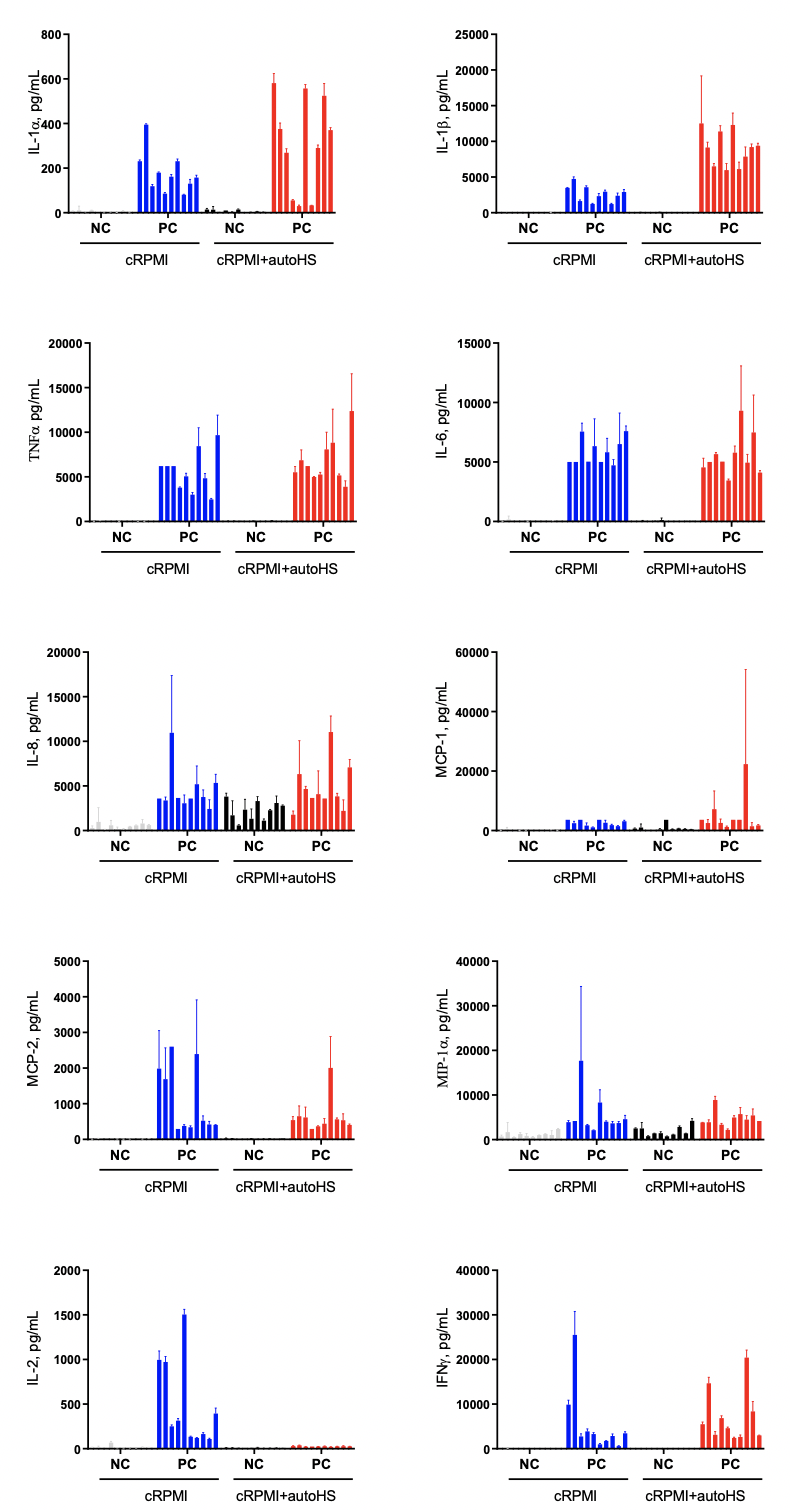
**Supplement**

**Supplementary Figure 1**. PBMC from 10 healthy donors were isolated from fresh, heparinized whole blood. The cells were cultured either in complete RPMI (cRPMI ) containing 10% heat-inactivated FBS, L-glutamine and pen/strep or in cRPMI supplemented with 10% autologous fresh human serum (autoHS) obtained from the same donors. Negative control (NC) was PBS; positive control (PC) was 20 ng/mL of *E.coli* K12 LPS. The incubation continues for 24 hours, after which the supernatants were analyzed by ELISA for the presence of cytokines. Each bar shows mean response and standard deviation (N=3).


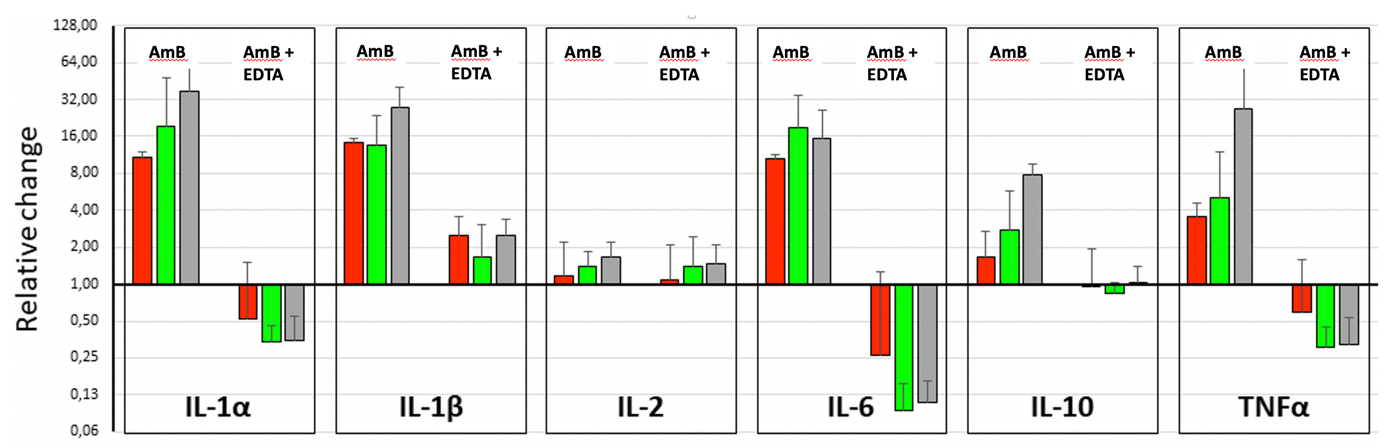


**Supplementary Figure 2.** Relative changes of IL-1α, β, IL-2, IL-6, IL-10 and TNFα levels in PBMC culture supernatants after 18 hours incubation with AmBisome alone (AmB) or together with 20 mM EDTA (AmB + EDTA). Similar measurements as shown in Fig. 4, using 3 donor PBMCs (red, gree and gray) that were different from the donors shown in Fig 4. The y axis shows the changes related to baseline (0 min), thus values below 1 (IL-1α, IL-6, TNFα) may imply inhibited spontaneous release of the cytokine. Values are mean +/- SD.
